# Are 90% of deaths from cancer caused by metastases?

**DOI:** 10.1002/cam4.2474

**Published:** 2019-08-08

**Authors:** Hanna Dillekås, Michael S. Rogers, Oddbjørn Straume

**Affiliations:** ^1^ Department of Clinical Science University of Bergen Bergen Norway; ^2^ Vascular Biology Program and Department of Surgery Boston Children's Hospital Harvard Medical School Boston MA USA; ^3^ Department of Clinical Medicine Centre for Cancer Biomarkers CCBIO University of Bergen Bergen Norway; ^4^ Department of Oncology and Medical Physics Haukeland University Hospital Bergen Norway

**Keywords:** cancer registry, cause of death, epidemiology, metastasis, survival

## Abstract

Numerous publications have stated that metastases are responsible for 90% of cancer deaths, but data underlying this assertion has been lacking. Our objective was to determine what proportions of cancer deaths are caused by metastases. Population‐based data from the Cancer Registry of Norway for the years 2005‐2015 was analyzed. We compared all deaths in the Norwegian population where a cancer diagnosis was registered as cause of death. Deaths caused by cancer, with and without metastases, were analyzed, by sex and tumor group. For solid tumors, 66.7% of cancer deaths were registered with metastases as a contributing cause. Proportions varied substantially between tumor groups. Our data support the idea that the majority of deaths from solid tumors are caused by metastases. Thus, a better understanding of the biology of metastases and identification of druggable targets involved in growth at the metastatic site is a promising strategy to reduce cancer mortality.

## Graphical abstract

In publications on cancer research, a common statement is that 90% of cancer deaths are caused by metastases, data underlying this assertion has been lacking. Using the nation‐wide, population based Norwegian Cancer Registry, we found that two of three of the deaths from solid tumors were registered with metastases as contributing cause of death, with substantial variation between tumor groups.

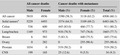

## 1 INTRODUCTION

Numerous publications, including some of ours, have stated that metastases are responsible for 90% of cancer deaths.^1^, ^2^, ^3^, ^4^ Clinical experience and biological reasoning would suggest that this is true. The statement is frequently used to justify a reinforced research focus on the biology of metastases. When looking into the references for this statement, however, it is unclear what data, if any, underlies this assertion. Scientific papers in which the statement appears refer to other papers that state the same thing. Those papers refer to yet others and after a few such rounds, the statement merely claims that most cancer deaths are caused by metastatic disease, without any references.^5^, ^6^, ^7^, ^8^ In our literature search, many of these reference pathways converge on a commentary by Sporn in The Lancet in 1996^5^ that actually does not touch upon the subject of what proportion of cancer deaths are caused by metastatic disease at all. Immense progress has been made in diagnosis and treatment of cancer since 1996, so even assuming that there was at some time point data supporting this statement, we need not conclude that this still holds true today. For example, childhood leukemia cure rates have increased substantially the last decades, thereby lowering the number of total cancer deaths registered as nonmetastatic disease. In breast cancer, improved diagnosis and treatment has led to a decline in total numbers of deaths as well as deaths from metastatic disease in relation to incidence, as incidence is increasing.^9^ Thus, it is not clear how improved diagnosis and treatment will affect the fraction of cancer deaths accompanied by metastasis (ie, #cancer deaths with metastasis/#cancer deaths). Improved care will presumably decrease both cancer deaths and cancer deaths from metastasis, but the effect on the fraction of deaths from metastasis is not obvious.

When considering deaths from cancer without metastatic disease as an underlying cause, a few different scenarios come to mind. First, we have cases where local tumors affect vital organs, such as airways, brain, heart, and liver. Second, systemic cancer treatment and its side effects can be fatal by multiple organ failure, bleeding due to thrombocytopenia, infections, interstitial pneumonitis, and tumor lysis syndrome to name a few. Third, surgical treatment and its potential complications can also have a fatal outcome. Fourth, tragically, some patients commit suicide after a cancer diagnosis, however, a recent publication on the SEER database demonstrated this to be more frequent in patients with metastatic disease.^10^

## 2 METHODS

As Norway has a tradition of robust and reliable registries, we set out to find the data to support or refute this truism. Reporting to the Cancer Registry of Norway and the Cause of Death Registry of Norway is mandatory for all physicians, and the latest published evaluation showed a 98.8% completeness of data.^11^ The registries record whether or not the cancer patient had been diagnosed with metastases, either before death or at autopsy if this was performed. We assumed that cancer death in a patient with metastatic disease would be caused by the metastases and not by the primary tumor, even in the rare occasions when the primary tumor was still intact. The latest available complete data are from 2015. In this year, a total of 10 944 cancer deaths were registered. Data are also available for primary site and sex. Site stratification is crucial, as some cancer forms are highly aggressive and associated with short survival even though they rarely metastasize, the best example probably being glioblastoma. We calculated the proportions of deaths from metastatic and nonmetastatic cancers in total and after excluding deaths from malignancies of lymphoid or hematopoietic origin (n = 1052), leaving 9892 cancer deaths in patients with solid tumors.

## 3 RESULTS

When looking at all cancer deaths per year from 2005 to 2016, there is surprisingly little change, from 10 575 in 2005 to 10 814 in 2016. The number of cancer deaths registered with metastasis as contributing cause of death on the other hand demonstrates a tripling, from 1953 (18.5%) in 2005 to 5575 (55.5%) in 2016. When looking into the details of the latest year (2015) with complete data on different tumor groups, our suspicion of substantial variation between tumor groups was confirmed. The proportions of all cancer deaths that were registered with metastasis ranged from 100% (testicular cancer, nose sinus) to 9.3% in central nervous system cancers (Table 1). For prostate cancer, the rate of cancer deaths registered with metastases as contributing cause was only 50.5%, a proportion that clinical experience would suggest to be almost all. Breast cancer incidence has been rising over the last decade, while deaths from breast cancer are declining. The proportion of deaths caused by metastatic disease, however, is remarkably stable (Table 2). For all solid tumors, the rate was 66.7%.

**Table 1:** Cancer deaths in Norway 2015

	All cancer deaths		Cancer deaths with metastases		
	Male	Female	Male (%)	Female (%)	Total (%)
All cancer	5810	4936	3390 (58.3)	3118 (63.2)	6508 (60.1)
Solid tumors^a^	5229	4493	3374 (64.5)	3109 (69.2)	6483 (66.7)
Colon	536	600	445 (83.0)	466 (77.7)	911 (80.2)
Lung/trachea	1169	973	918 (78.5)	747 (76.8)	1665 (77.7)
Breast	6	583	5 (83.3)	440 (75.5)	445 (75.6)
Ovary	0	282	0	255 (90.4)	255 (90.4)
Prostate	1034	0	519 (50.2)	0	519 (50.2)
CNS	199	165	25 (12.6)	9 (5.5)	34 (9.3)

a Excluding lymphomas and hematologic malignancies.

**Table 2:** Breast cancer in Norway

Year	Incidence	Cancer deaths	With metastases (%)	Cancer deaths by incidence	With metastases by incidence
2007	2729	697	529 (75.9)	25.5%	19.4%
2010	2849	663	509 (76.8)	23.3%	17.9%
2015	3422	589	445 (75.6)	17.2%	13.0%

## 4 DISCUSSION

Having read the statement that metastases are responsible for 90% of deaths from cancer in a number of papers, that did not seem to have supporting data, neither in themselves nor in references, we sought to evaluate this truism. The reasons it has become such a widely accepted notion are worth some consideration. First, experience from the clinic is consistent with the proportion being in this range. Second, biological reasoning would also suggest that very few localized tumors are capable of killing the patient. Third, since it has been written in seminal papers by some of the most prominent cancer scientists, it has been given substantial weight. When more and more papers have written it, the “evidence” of it being true seems to accumulate, even in the absence of supporting data. In this nationwide, population‐based registry study, we found that 66.7% of cancer deaths in solid tumors were caused by metastases. The proportion of deaths attributed to metastatic disease has been increasing dramatically over the last decade. It seems unlikely that this reflects clinical reality and is more likely due to increased focus on correct registration in death certificates. Based on clinical experience, these numbers still seem far too low, and interviews with staff from the registry confirmed that whereas synchronous metastases (ie, metastases diagnosed at the same time as the primary tumor) is well reported, metachronous metastases (metastases discovered at some later time point) are underreported. As described above, registration rates seem to be improving, but still, based on Norwegian registry data, we unfortunately cannot determine the precise proportion of cancer deaths caused by metastatic disease. The improving registration rates over the past decade, however, give reason to hope that we will be able to answer this question with robust, reliable data in the future. Nevertheless, our data support the idea that the majority of deaths (at least 2/3) from solid tumors are caused by metastases. Thus, a better understanding of the biology of metastases and identification of druggable targets involved in growth at the metastatic site is a promising strategy to reduce cancer mortality.

## DISCLAIMER

This work uses data from the Norwegian Cancer Registry. Interpretation and reporting of this data is the sole responsibility of the authors, and has not been subject of approval from the Norwegian Cancer Registry.
